# Large language models and rheumatology: are we there yet?

**DOI:** 10.1093/rap/rkae119

**Published:** 2024-09-18

**Authors:** Diego Benavent, Alfredo Madrid-García

**Affiliations:** Rheumatology Department, Hospital Universitari de Bellvitge, Barcelona, Spain; Medical Department, Savana Research SL, Madrid, Spain; Grupo de Patología Musculoesquelética, Hospital Clínico San Carlos, Instituto de Investigación Sanitaria San Carlos (IdISSC), Madrid, Spain

**Keywords:** large language models, rheumatology, chatGPT, natural language processing, artificial intelligence

## Abstract

The last 2 years have marked the beginning of a golden age for natural language processing in medicine. The arrival of large language models (LLMs) and multimodal models have raised new opportunities and challenges for research and clinical practice. In rheumatology, a specialty rich in data and requiring complex decision-making, the use of these tools may transform diagnostic procedures, improve patient interaction and simplify data management, leading to more personalized and efficient healthcare outcomes. The objective of this article is to present an overview of the status of LLMs in the field of rheumatology while discussing some of the challenges ahead in this area of great potential.

## Graphical abstract

**Graphical Abstract:**
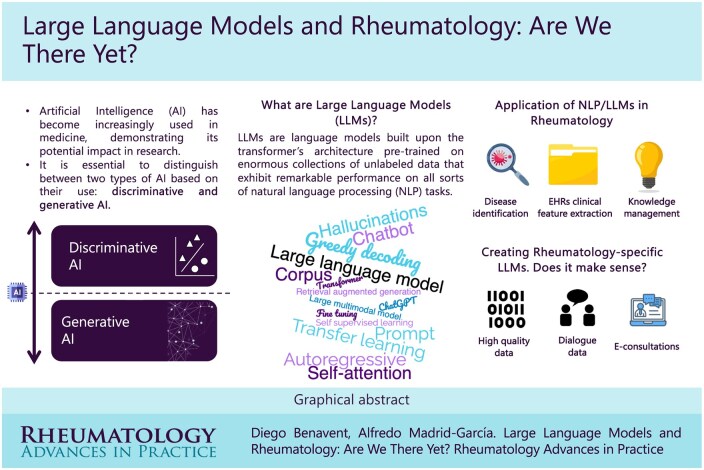

Key messages

- Large language models (LLMs) may support rheumatology research and practice by summarizing texts, generating code or answering questions.
- Despite the potential impact of LLMs, guidelines concerning their ethical application have not yet been published.
- Electronic health records and e-consults can be a key asset for training rheumatology-specific LLMs.

## Introduction

Artificial intelligence (AI) has become increasingly used in medicine, demonstrating its potential impact in research in several recent projects [1]. Notably, the introduction of platforms such as ChatGPT has marked a turning point in the adoption of this technology [2, 3]. In rheumatology, the revolution brought about by AI is beginning to show promise, offering opportunities for research and clinical practice [4].

Following this evolution, it is essential to distinguish between two types of AI based on their use, namely discriminative AI and generative AI. Discriminative AI focuses on the development of models designed to learn and progressively improve performance across various tasks, such as data classification and prediction, through repeated data analysis using machine learning or deep learning algorithms [5, 6]. Discriminative AI has been incorporated into rheumatology research for some years, showing examples on disease subgroup stratification, improved diagnoses and prognosis predictions [7–9]. On the other hand, generative AI, can create new content based on training data, including text generation, code automation or image creation. This marks an expansion of our capabilities, as generative AI can improve the organization of information, aiding in scientific manuscript writing, medical report generation and helping in the creation of decision support tools.

Input data for both discriminative and generative AI algorithms can be derived from large structured databases and other sources, such as imaging or free text. Initially, structured databases yielded some studies in rheumatology. Later on, imaging provided data for many studies, for example demonstrating superior performance in detecting erosions in sacroiliac joint computed tomography scans in spondyloarthritis (SpA), compared with radiologist evaluations [10]. Notwithstanding, the analysis of free text to extract data has shown great advances in recent years [9, 11–14].

Indeed, most clinical and research information is presented in unstructured formats such as text, posing challenges for data analysis and collection. In this context, natural language processing (NLP) emerges as a critical tool, bridging the gap between linguistics and analysis and facilitating the task of data collection from free text [15]. These non-structured data sources, including real-life data from clinical practice, hold promise for exploring patient comorbidities, adverse events and resource utilization. NLP techniques have been successfully applied to accurately identify patients with rheumatoid arthritis (RA) or SpA from clinical records and to extract mentions of outcome measures in RA with high sensitivity and positive predictive value (PPV) [16, 17].

Since the development of the transformer technology, the evolution of NLP has been very rapid, particularly with the emergence of large language models (LLMs) [18]. LLMs are advanced AI systems designed to understand, generate and interact with human language [19]. These models are trained on vast amounts of text data based on the transformer technology, allowing them to understand the context and complexities of language. While traditional NLP techniques often relied on rule-based systems that required extensive feature engineering and domain-specific knowledge, LLMs can perform complex language tasks with minimal human intervention. Therefore, LLMs can generate coherent and contextually relevant text based on this input they receive. In this regard, LLMs can answer questions, summarize text, translate and generate code, showing versatility across various domains. In the context of healthcare, this has many use cases, from analysing medical literature to assisting in clinical decision-making and patient communication. LLMs offer a promising tool for enhancing both research and clinical practice.

To create a comprehensive summary of the state-of-the-art in NLP and LLMs in rheumatology, we performed an extensive electronic search in Medline, Embase and arXiv for English-language sources from inception to May 2024. We employed a range of free-text terms including, but not limited to: ‘Large language models’, ‘Natural language processing’, ‘LLM’, ‘NLP’, ‘NLP AND electronic health records’, ‘ChatGPT’, ‘LLMs in healthcare’, ‘Artificial intelligence AND Rheumatology’, ‘LLM AND rheumatology’, ‘NLP AND Rheumatology’, ‘ChatGPT AND rheumatology’. The references obtained were systematically managed using Mendeley to ensure efficient organization and retrieval. Furthermore, we conducted a manual search by examining the references cited in the included studies and technical computer science books. Priority was given to seminal references or those published within the last 2 years.

## What are large language models?

### Technical definition

LLMs are **language models** built upon the **transformer’s architecture** (i.e. neural networks with millions of parameters), **pre-trained** on enormous collections of unlabelled data (usually coming from the web), and following **self-supervised learning** approaches; that exhibit remarkable performance on all sorts of natural language tasks.

### Understanding the definition

To fully understand what a LLMs is, different concepts should be introduced:

- ***What is a language model?*** Language models are models that assign **probabilities** to upcoming words or sequence of words from neighbouring words. Specifically, a language model estimates how likely different words or sequences of words are to occur. In simpler terms, language models are capable of predicting the next word based on the previous ones.
- ***What is a transformer?*** Transformers are deep neural network models (i.e. artificial neural networks that contain a deep stack of hidden layers) made up of stacks of transformer blocks (i.e. multilayer networks) that map sequences of input vectors to sequences of output vectors of the same length [20]. The aim of these layers is to build increasingly complex and context-enriched interpretations of the meanings of input words [21]. Eventually, a representation for every word that incorporates context-specific information (i.e. surrounding words) is generated. To create these contextualized representations, transformers rely on **self-attention mechanisms** which capture the dependencies between the words in a sentence. In this scenario, attention is understood as a neural network layer whose ultimate goal is to learn long-range global features, deciding which components of the input sequence contribute the most to the output, assigning a different amount of weight to each element in a sequence. This architecture has gained relevance and has surpassed others (e.g. recurrent neural networks), due to several factors such as the mitigation of vanishing and exploding gradients issues (i.e. gradients become extremely small or large, halting the training or causing unstable training) by removing recurrent connections [22]. Moreover, the training is achieved with fewer steps, parallelization is easier, and longer-range patterns are better captured. In LLMs, self-attention is limited to **causal attention** (i.e. only previous words are considered), and the transformer architecture, normally, only comprises the **decoder** [23], from the encoder–decoder module (i.e. focusing solely on generating output based on the input and previously generated context, without needing to encode the input into a separate intermediate representation). The text is generated in an **autoregressive** manner (i.e. decoding from left to right).
- ***How are transformers taught to be LLMs?*** A transformer is trained in two steps. **Pretraining** is the initial phase of training the transformer model before it undergoes further **fine-tuning**. During pretraining, the model learns the general structure of a language (i.e. language patterns, vocabulary usage and so on) by exposing it to vast amount of textual unannotated data (i.e. usually millions of **tokens**, where a token is a word or parts of words, depending on the **tokenizer**), commonly retrieved from the web, in an unsupervised manner. More specifically, **self-supervised learning** is applied. With this approach, for each time step t, the algorithm asks the model to forecast the subsequent word, this is the model learns to predict some parts of the input data from other parts of the same data, without requiring any human-labeled data. Thanks to pretraining, the model learns to generalize. On the other hand, fine-tuning is the task of further training the model to perform downstream tasks with labelled data. This is, once the model has learned the language, it is trained to perform specific tasks with specific data of the desired application. Thanks to fine-tuning the model learns how to perform specific tasks, such as question-answering or named entity recognition. Other adaptations to train the LLMs, in addition to fine-tuning, exist such as *Reinforcement Learning from Human Feedback* [24] or *Retrieval-Augmented Generation* [25].
- ***What is the relationship between Generative AI and LLMs?*** Generative AI is a broad category of AI systems designed to generate new content (e.g. text, images, music, video), based on their training data. LLMs are a subset of generative AI focused specifically on understanding and generating human-like text. Essentially, LLMs are a type of generative AI that specializes in text generation and natural language tasks. When LLMs can accept more than one type of input and generate outputs that are not limited to the type of data entered, they are called large multimodal models (LMMs) [26].

### Other relevant concepts

e) ***What is ChatGPT?***ChatGPT is a neural conversational agent (i.e. chatbot), design by OpenAI [27], that mimics the informal human-human conversations, which is designed to maintain longer and more unstructured conversations than a typical conventional dialogue system. It can be seen as a specific implementation of a LLM trained with lots of dialogue data.
f) ***Why do chatbots based on LLMs produce different outputs even though the input is similar?***Because the generation of the next word is not based on the most likely word given the context (i.e. **greedy decoding**), which would produce generic and deterministic texts, but on sampling methods. With sampling methods, words are randomly chosen considering their probabilities in such a way that words with higher probabilities have higher chances to be selected. In LLMs, the probability of each word is conditioned on previous choices.
g) ***What is a prompt?***A prompt is an instruction or input given to a LLM that guides its output. It serves multiple purposes, including enforcing rules, automating processes, ensuring the quality and quantity of the generated content, and customizing interactions with the model [28, 29]. This concept is behind the prompt engineering notion (i.e. crafting of precise, task-specific instructions in natural language, intended to program or guide LLMs to achieve a specific goal or output). Recommendations, guides and good principles for prompt engineering have been published elsewhere [30–33]. In rheumatology, the relevance of prompt engineering has been highlighted in [34].
h) ***What are the current challenges of LLMs?***LLMs present a myriad of challenges [35]. However, **hallucinations** are particular problematic [36]. Hallucinations are commonly defined as information produced by LLMs that might seem accurate but is actually misleading. This can be a significant issue because they sometimes include partially accurate data, leading users to mistakenly believe that all the information provided by the model is accurate, resulting in misunderstandings or the propagation of misinformation. Nowadays, mitigation strategies are being developed [37].
i) ***What types of data are used for fine-tuning LLMs?*** Instruction data is commonly used for fine-tuning LLMs (i.e. **instruction fine-tuning)**. Briefly, instruction data is a collection of data specifically designed for training and testing models that perform tasks related to understanding and generating human-readable instructions. These datasets often include pairs of input–output examples where the input is a set of instructions in natural language or a structured format, and the output is the desired action or outcome corresponding to those instructions. When training LLMs, fine-tuning with instructions involves training the model by showcasing examples that illustrate its desired responses to particular instructions. In medicine, some general-purpose instruction and question-answering datasets have been built, such as MedAlign [38], MedInstruct-52k [39] or MedMCQA [40]. Comprehensive lists of public datasets for training LLMs are regularly updated in GitHub [41]. More than 400 datasets for training these models have recently been explored in [42].

## Classification of LLMs

Table 1 provides a classification of LLMs, outlining their characteristics across several dimensions to support the main objective of understanding the diversity and specificity of LLMs in various applications. LLMs are based on the number of parameters, indicating the complexity, ranging from billions to trillions of variables. LLMs can be classified into proprietary public (freely available general-purpose models), proprietary private (domain-specific models using private data), and open source. Additionally, LLMs can be categorized by modality (unimodal like ChatGPT 3.5, trained on text only, and multimodal like ChatGPT 4, integrating text, images, and audio), domain-specificity (general-purpose versus domain-specific models) and language capabilities (monolingual primarily in English versus multilingual models like ChatGPT). These classifications enhance the understanding of LLMs’ capabilities and accessibility, essential for deploying them effectively across varied applications.

**Table 1.** Classification criteria of LLMs

Criteria	Description	
Number of parameters	Number of variables in the neural network. This correlates with the LLMs complexity. Modern models range from billions to trillions of variables. LLMs with more parameters generally offer advanced understanding and generation capabilities.	
Availability/accessibility	Proprietary public	LLMs freely available to the public. These LLMs are usually general-purpose models (e.g. ChatGPT 3.5, Google Gemini Pro [43], Claude 3 Sonnet [44]).
	Proprietary private	LLMs not accessible to the public, usually trained using private data, and designed to excel in specific objectives. These models are often domain-specific, after having undergone a fine-tuning process. Model such as Med-PaLM 2 [45] or AMIE [46], could be considerate private LLMs. This category may also encompass models that are geographically restricted due to regulatory and legal considerations; or general-purpose models available to the public but behind a paywall (e.g. ChatGPT 4, Google Gemini Ultra or Claude 3 Opus).
	Open source	According to some authors, LLMs adhering to *The Open Source Definition* [47] are considered open source models. However, other authors relax this definition to include in this category models such as LLaMA [48]. Although efforts are being made, it is not common to find LLMs with the training code, the training data, intermediate checkpoints, technical reports, and the final model weights available [49]. Falcon [50], LLaMA or Vicuna [51] are some examples of models considered open-sourced by the community.
Modality	Unimodal	LLMs trained on a single data modality, such as text (e.g. ChatGPT 3.5, LLaMA 2).
	Multimodal	LLMs able to process and integrate multiple types of data such as text, images, and audio (e.g. ChatGPT 4, Claude 3 Sonnet) [52].
Domain-specificity	General-purpose	Models intended to handle a wide variety of topics and tasks, without a particular specialization. They are suitable for most users in their daily activities. Most of today’s proprietary LLMs are general-purpose.
	Domain-specific	Models that attempt to solve specific tasks (e.g. patients’ questions). Domain-specific models may not necessarily be trained exclusively with data from their specialized domain. Instead, they can start as general-purpose models and later undergo fine-tuning with domain-specific data. In [53], a survey of LLMs in medicine is provided. Some examples of healthcare LLMs are Hippo [54], BioMistral [55] or MEDITRON [56]. In [57], a leaderboard of open medical LLMs is shown.
Language	Monolingual	Models designed to understand, generate, and interact with text in a single specific language. However, the performance of LLMs on different languages may vary, since the bulk of conversational training data is primarily in English, most LLMs are developed to be monolingual [58].
	Multilingual	Models designed to understand, generate, and interact with text in multiple languages. These models are trained on vast amounts of text data from a wide range of languages (e.g. ChatGPT, Claude, Gemini). Currently, several national agencies are working on creating models that support co-official languages within the same country.

## GPT-4 Omni

GPT-4 Omni is the latest LLM developed by OpenAI, GPT-4 Omni (GPT-4o), that was presented during the writing of this manuscript. GPT-4o is a multimodal model capable of processing and reasoning across audio, image, video and text inputs in real time, all with minimal latency. According to developers, this model can respond to audio inputs with a similar response time comparable to that of humans. This creates a spectrum of opportunities in medicine, and also in rheumatology, in the following domains:

- Medical transcription: traditional transcription approaches may encounter difficulties with the complexities of medical terminology, resulting in inaccuracies and inefficiencies. Moreover, the transcription is usually conducted in plain text. However, with LMMs new capabilities could be unlocked such as structuring the data in tabular format during the transcription or to conduct real-time translations.
- Enhancing accessibility for visually impaired patients and low-literacy patients: patients with visual impairments, such as uveitis patients could be benefitiated of educational materials about their disease in audio format [59]. Similarly, models capable of generating audio and maintaining conversations could enhance health equity by closing the education gap in under-resourced areas where access to written materials may be limited.
- Triage and citation: although prior iterations of LLMs have not shown consistent results across studies when using textual data from the emergency departments (EDs) to classify clinical acuity [60, 61], newer models such as GPT-4o could prove beneficial in outpatient settings. To begin with, already followed patients could seek advice about new concerns or symptoms during non-working hours. An LLM could evaluate the clinical risk and send an alert to the physician or schedule an appointment, in addition to providing clinical advice in a more immediate way than by sending messages in textual format. Nonetheless, validation of these models is yet to be shown in clinical studies.

## Application of NLP/LLMs in rheumatology

As it has been discussed, NLP allows to interpret and analyse free text [62]. This capability is enhanced using LLMs, which employ NLP for specific tasks such as text generation. Rheumatology is a discipline confronted with a wide array of disorders that display substantial variation in clinical manifestations, which makes AI, and particularly the analysis of free text through these technologies, very promising [63].

**Figure 1.**
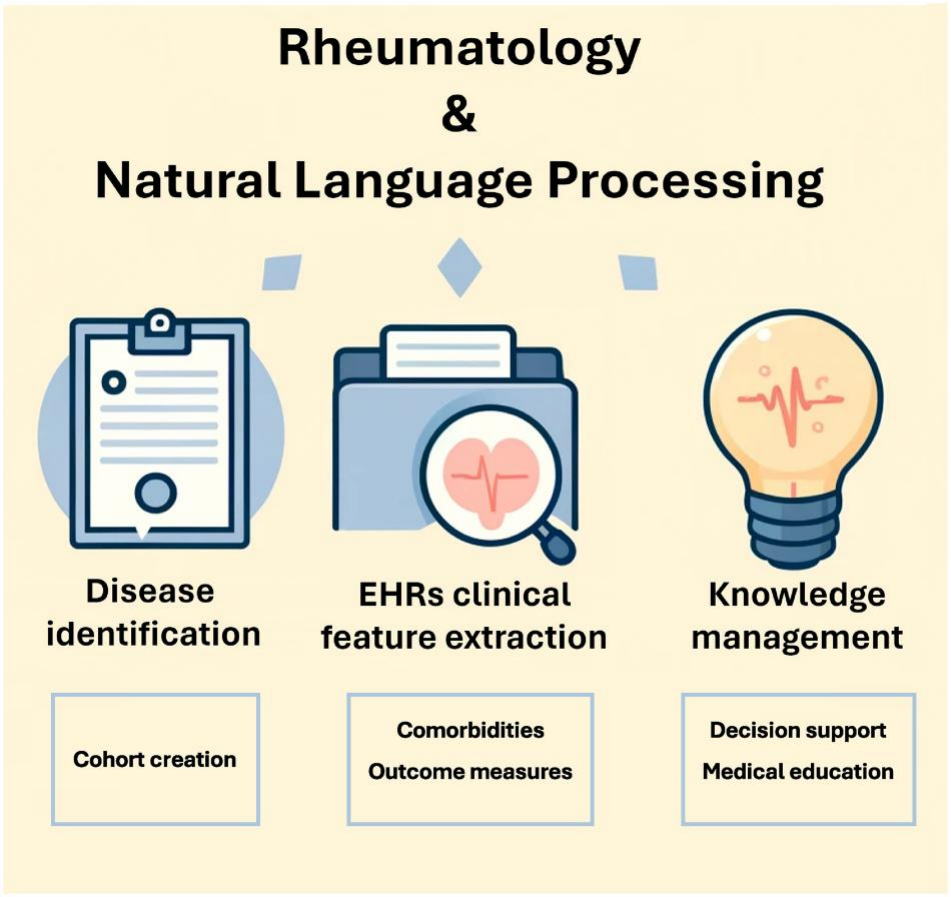
Applications of natural language processing in rheumatology

NLP facilitates the analysis of electronic health records (EHRs), covering hundreds to millions of clinical notes, for various purposes including identifying diseases and assessing disease characteristics. Besides, it can help in question answering and knowledge management, serving as a potential useful tool for supporting clinical decision-making (Fig. 1).

### Disease identification

In the domain of disease diagnosis and classification across rheumatic diseases, numerous studies highlight the efficacy of NLP to extract this information of EHRs. For instance, different algorithms have demonstrated the utility of in identifying axial spondyloarthritis (axSpA) or psoriatic arthritis (PsA), achieving a good sensitivity and specificity [64, 65]. Recent research has effectively utilized an AI tool incorporating NLP to detect ANCA-associated vasculitis, reporting sensitivity rates between 96.3% and 98.0% [66]. This growing body of evidence supports the adoption of NLP technologies in identifying rheumatic diseases.

### EHRs clinical features extraction

Several studies extend beyond disease identification to extract other clinical information from EHRs. For example, an NLP pipeline was utilized within the Rheumatology Informatics System for Effectiveness (RISE) registry to extract data from free-text outpatient rheumatology notes [67]. This approach demonstrated NLP’s capacity for analysing extensive clinical data, retrieving RA outcomes from over 34 million notes with a sensitivity of 95%, an F1-score of 91% and a PPV of 87%. The RA-WILD study effectively identified the clinical characteristics of patients with RA with interstitial lung disease (RA-ILD) from a dataset exceeding 64 million EHRs, achieving a precision of over 0.7 for most of the assessed variables [68]. This research showed that RA-ILD patients exhibited greater vulnerability, with higher comorbidity and inflammatory burden compared with RA patients without ILD.

Certain studies have concentrated on specific components reported on the EHRs. The SpAINET study targeted the assessment of disease activity, revealing that only approximately one-third of axSpA and one-sixth of PsA patients underwent disease activity assessments [17]. England *et al*. [69] developed an NLP tool to extract Forced Vital Capacity from EHRs, demonstrating a high correlation (*r* = 0.94) with values from pulmonary function tests. Lin *et al*. [70] applied NLP to identify methotrexate-induced liver toxicity in patients with RA, achieving a PPV of 0.76.

Another study focused on improving the detection of gout flares in the ED, using both rule-based and advanced NLP algorithms [71]. This research demonstrated that analysing chief complaints alone could effectively identify gout flares with high accuracy, potentially enhancing gout flare management by enabling prompt identification and referral during ED visits. These advancements further exemplify NLP’s transformative impact on rheumatology and patient management.

### Knowledge management

A recent study compared the performance of GPT-4, Claude (versions 1.3 and 2), and Bard in rheumatology-specific trivia, highlighting their varying capabilities and the potential impacts of their features on medical practice [72]. GPT-4 and Claude 2 demonstrated superior performance, correctly answering 81% of the questions. Another study evaluated the effectiveness of GPT-4 in the context of rheumatology education, specifically their ability to answer questions from the Spanish MIR medical training exams, on their accuracy and clinical reasoning. GPT-4 showed and impressive accuracy of 93.71% and clinical reasoning of 4.7 on a 5-point Likert scale [73]. Additionally, a recent study assessed ChatGPT-4’s diagnostic accuracy for inflammatory rheumatic diseases (IRDs), comparing it to rheumatologists in a controlled dataset [74]. ChatGPT-4 identified the correct top diagnosis in 35% of cases versus 39% for rheumatologists and placed the correct diagnosis within the top three 60% of the time, compared with 55% for rheumatologists. ChatGPT-4 excelled in IRD-positive cases, listing the correct top diagnosis in 71% of cases versus 62% for rheumatologists and within the top three 86% of the time versus 74%. These findings suggest that NLP has the potential to augment the diagnostic process in rheumatology, potentially serving as a clinical decision support tool.

## Creating rheumatology-specific LLMs. Does it make sense?

An important question that requires careful consideration is whether it is necessary to develop domain-specific LLMs for each medical specialty, or whether it would be more advantageous to train a comprehensive model encompassing the entire healthcare sector, such as Google is doing with Med-PaLM. By focusing on a narrower domain, these models could potentially achieve higher accuracy. However, omitting the global medical knowledge and the physiological interactions between the different specialties/systems could limit the models’ understanding and ability to provide holistic patient care. The answer to the question posed is not a simple one. This trade-off should be further explored, by training and comparing specific-specialty-domain LLMs, and the answer will probably depend on the use case.

As of now, there are not publicly available LLM specifically designed for rheumatology, contrary to what has been observed in other domains such as oncology. In a recent study, the authors collected more than 180k oncology-related conversations from 13 doctor–patient platforms [75]. This was done by filtering dialog data collected from different online medical platforms, using oncology-related keywords. Afterwards, researchers conducted multiple rounds of fine-tuning on LLaMA, an open-source LLM developed by Meta, using the refined dialogue data. Hyperparameter selection and model validation phases were conducted to ensure optimal performance and accuracy of the model. Both the model, the data used for training it, and the best hyperparameters were made public.

The first challenge in developing domain-specific models lies in obtaining a sufficient quantity of accessible, high-quality data from the relevant domain. To the best of our knowledge, this kind of data in rheumatology is scarce or non-existent. Additionally, this web data may be subject to copyright protections, rendering it unusable for commercial purposes. Recent efforts are being made to solve this. For instance, as it has recently done by building the first linguistic corpus, *RheumaLpack*, in the field of musculoskeletal diseases that could be used for training LLMs [76]. However, this corpus lacks instruction data, so this information should be recovered from somewhere else. For instance, e-consults could be a valuable data source for generating specific training data for LLMs. Centres that have been conducting e-consultations and which are authorized to use such data, would be positioned with a greater competitive advantage to develop the first rheumatology-LLM [77, 78]. It appears inevitable that this type of data will be utilized to train specialized models for specific medical specialties in the upcoming years.

## Current challenges, ethical dilemmas and how to not misuse LLMs

The rise of LLMs has sparked several ethical challenges and considerations [79]. The speed at which LLMs have been adopted has not been matched by efforts to establish ethical guidelines or principles for their proper use. According to the *Stanford AI Index Report 2024*, one of the top 10 takeaways is that robust and standardized evaluations for LLM responsibility are seriously lacking [80]. One of the most recent attempts has been made by the WHO, which earlier this year published its guidance on large multi-modal models (LMMs) [26]. This guidance, which is closely related to a previous one [81] and which is complemented by different related publications [82–84], highlights the necessity of international efforts and collective development of international rules for the proper governance of LLMs. The report even discusses the possibility of regulating LLMs with the same stringent criteria used for nuclear weapons.

The European Union’s position on general-purpose AI models, such as LLMs is reflected in the *EU AI Act*, the world’s first major legislation on AI which will enter into force in 2026 [85]. Under this legislation, models that possess high-impact capabilities and present a great ‘systemic risk’ are classified into a more stringent regulatory category. On the other hand, *The Executive Order on AI* promoted in the USA pursues to create the necessary guidelines to safely exploit AI’s potential while reducing its associated risks. As a first step, the National Institute of Standards and Technology (NIST) has published four preliminary documents aimed at enhancing the safety, security, and reliability of AI systems. For instance, NIST AI 600-1 details 12 risks and actions for mitigation risks specific to Generative AI, such as dangerous recommendations or bias.

**Figure 2.**
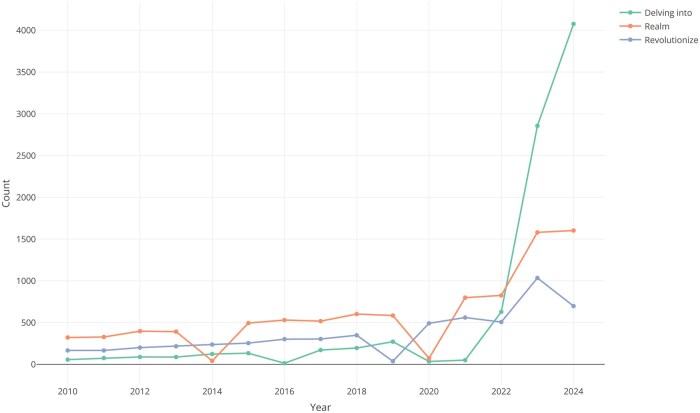
Appearance of common words used by ChatGPT in PubMed abstracts or titles

The medical and research community holds mixed views regarding the use of LLMs. On the one hand, some researchers encourage the use of LLMs. As an example, the *New England Journal of Medicine AI* published an editorial article in which they encouraged the use of LLMs: *We believe that the use of LLM tools can help scientists enhance the quality of their scientific work and democratize both the creation and consumption of scientific knowledge* [86]. On the other hand, some researchers express concerns about the implications of LLMs in the medical field, arguing that these technologies pose a threat to the profession [87], and that this kind of technology is not ready for clinical use [88]. In an effort to adapt to the new reality, driven by LLMs, most scientific journals and publishers have developed AI policies where authors are forced to provide details of any AI used in the manuscript elaboration. This has complemented by the emergence of new reporting guidelines such as CANGARU [89], CHEER [90], CHART [91] or the update of previous ones, such as MI-CLAIM [92]. These efforts have been partly driven by the rising usage of terms and expressions that, until recently, were uncommon in the scientific literature and are now widely recognized as hallmarks of ChatGPT*: delve, leverage, dive, deep, harness, foster, captivate, revolutionize* or *realm* [93]. An example of this increase in the use of some terms is shown in Fig. 2. Moreover, in some high-profile cases, the unethical use of these models, where their used was undeclared, fraudulent, or dishonest (e.g. appearance of ‘*Regenerate response*’ or ‘*As an AI language model, I…*’ sentences; fake images [94], or fake references) has contributed to the retraction of scientific articles [95]. This misuse in academia has recently become a topic of discussion and is likely to pose additional challenges in the coming years [96, 97].

There are not currently recommendations or points to consider on the ethical use of LLMs in rheumatology. However, as the field continues to evolve, it is to be expected that agreement will be reached on its use. According to Gartner’s 2023 Hype Cycle for Artificial Intelligence, Generative AI is in the peak of inflated expectations with the plateau of productivity to be reached in 5–10 years [98]. As this technology matures and reaches widespread adoption, it will be essential to evaluate its impact on clinical practice in rheumatology and re-analyse the ethical challenges.

The potential of LLMs in rheumatology is vast, offering multiple benefits and posing significant challenges. These models can revolutionize research by summarizing a great amount of medical literature and generating hypotheses for further investigation. LLMs also hold promise for advancing diagnostic processes by rapidly analysing complex clinical data and improving patient interactions through more accurate and personalized communication. However, their integration into research and clinical practice must overcome hurdles such as ensuring data privacy, addressing ethical concerns related to transparency, and managing the risk of generating misleading or inaccurate information. Implementing continuous evaluations and creating recommendations will be vital in maximizing the benefits of LLMs while addressing potential risks in rheumatology.

## Data Availability

Data sharing not applicable to this article as no datasets were generated or analysed during the current study.

## References

[1] Sahni NR , Carrus B. Artificial intelligence in U.S. health care delivery. N Engl J Med 2023;389:348–58. 37494486 10.1056/NEJMra2204673

[2] ChatGPT is the Fastest Growing App in the History of Web Applications. https://www.forbes.com/sites/cindygordon/2023/02/02/chatgpt-is-the-fastest-growing-ap-in-the-history-of-web-applications/?sh=405c0d25678c (14 December 2023, date last accessed).

[3] Cron RQ , Chatham WW. The rheumatologist’s role in COVID-19. J Rheumatol 2020;47:639–42. 32209661 10.3899/jrheum.200334

[4] Hügle M , Omoumi P, van Laar JM, Boedecker J, Hügle T. Applied machine learning and artificial intelligence in rheumatology. Rheumatol Adv Pract 2020;4:rkaa005. 32296743 10.1093/rap/rkaa005 PMC7151725

[5] Morales-Ivorra I , Narváez J, Gómez-Vaquero C et al Assessment of inflammation in patients with rheumatoid arthritis using thermography and machine learning: a fast and automated technique. RMD Open 2022;8:e002458. 35840312 10.1136/rmdopen-2022-002458 PMC9295660

[6] Lee H , Tajmir S, Lee J et al Fully automated deep learning system for bone age assessment. J Digit Imaging 2017;30:427–41. 28275919 10.1007/s10278-017-9955-8 PMC5537090

[7] Joo YB , Baek IW, Park YJ, Park KS, Kim KJ. Machine learning-based prediction of radiographic progression in patients with axial spondyloarthritis. Clin Rheumatol 2020;39:983–91. 31667645 10.1007/s10067-019-04803-y

[8] Lee S , Kang S, Eun Y et al Machine learning-based prediction model for responses of bDMARDs in patients with rheumatoid arthritis and ankylosing spondylitis. Arthritis Res Ther 2021;23:254. 34627335 10.1186/s13075-021-02635-3 PMC8501710

[9] Larrainzar-Garijo R , Fernández-Tormos E, Collado-Escudero CA et al Predictive model for a second hip fracture occurrence using natural language processing and machine learning on electronic health records. Sci Rep 2024;14:532. 38177650 10.1038/s41598-023-50762-5 PMC10766963

[10] Castro-Zunti R , Park EH, Choi Y, Jin GY, Ko S. B. Early detection of ankylosing spondylitis using texture features and statistical machine learning, and deep learning, with some patient age analysis. Comput Med Imaging Graph 2020;82:101718. 32464565 10.1016/j.compmedimag.2020.101718

[11] Román Ivorra JA , Trallero-Araguas E, Lopez Lasanta M et al Prevalence and clinical characteristics of patients with rheumatoid arthritis with interstitial lung disease using unstructured healthcare data and machine learning. RMD Open 2024;10:e003353. 38296310 10.1136/rmdopen-2023-003353 PMC10836356

[12] Sheikhalishahi S , Miotto R, Dudley JT et al Natural language processing of clinical notes on chronic diseases: systematic review. JMIR Med Inform 2019;7:e12239. 31066697 10.2196/12239 PMC6528438

[13] Zhao SS , Hong C, Cai T et al Incorporating natural language processing to improve classification of axial spondyloarthritis using electronic health records. Rheumatology (Oxford) 2020;59:1059–65. 31535693 10.1093/rheumatology/kez375 PMC7850056

[14] Humbert-Droz M , Izadi Z, Schmajuk G et al Development of a natural language processing system for extracting rheumatoid arthritis outcomes from clinical notes using the National Rheumatology Informatics System for Effectiveness Registry. Arthritis Care Res (Hoboken) 2023;75:608–15. 35157365 10.1002/acr.24869

[15] Demner-Fushman D , Chapman WW, McDonald CJ. What can natural language processing do for clinical decision support? J Biomed Inform 2009;42:760–72. 19683066 10.1016/j.jbi.2009.08.007 PMC2757540

[16] Humbert-Droz M , Izadi Z, Schmajuk G et al Development of a natural language processing system for extracting rheumatoid arthritis outcomes from clinical notes using the National Rheumatology Informatics System for Effectiveness Registry. Arthritis Care Res (Hoboken) 2023;75:608–15. 35157365 10.1002/acr.24869

[17] Benavent D , Muñoz-Fernández S, De la Morena I et al; SAVANA Research Group. Using natural language processing to explore characteristics and management of patients with axial spondyloarthritis and psoriatic arthritis treated under real-world conditions in Spain: SpAINET study. Ther Adv Musculoskelet Dis 2023;15:1759720X231220818. 10.1177/1759720X231220818 PMC10749530 38146537

[18] Venerito V , Bilgin E, Iannone F, Kiraz S. AI am a rheumatologist: a practical primer to large language models for rheumatologists. Rheumatology (Oxford) 2023;62:3256–60. 37307079 10.1093/rheumatology/kead291 PMC10547503

[19] Singhal K , Azizi S, Tu T et al Large language models encode clinical knowledge. Nature 2023;620:172–80. 37438534 10.1038/s41586-023-06291-2 PMC10396962

[20] Vaswani A , Shazeer N, Parmar N et al Attention is all you need. 2023. https://arxiv.org/abs/1706.03762 (24 May 2024, date last accessed).

[21] Jurafsky D , Martin JH. Speech and language processing: an introduction to natural language processing, computational linguistics, and speech recognition. 3rd edn. 2023. https://web.stanford.edu/∼jurafsky/slp3/ (24 May 2024, date last accessed).

[22] Géron A. Hands-on machine learning with Scikit-Learn, Keras, and TensorFlow. Sebastopol, California, USA: O’Reilly Media, Inc., 2022.

[23] Zhao WX , Zhou K, Li J et al A survey of large language models. 2023. https://arxiv.org/abs/2303.18223 (24 May 2024, date last accessed).

[24] Ouyang L , Wu J, Jiang X et al Training language models to follow instructions with human feedback. 2022. https://arxiv.org/abs/2203.02155 (24 May 2024, date last accessed).

[25] Lewis P , Perez E, Piktus A et al Retrieval-augmented generation for knowledge-intensive NLP tasks. 2021. https://arxiv.org/abs/2005.11401 (24 May 2024, date last accessed).

[26] World Health Organization. Ethics and governance of artificial intelligence for health Guidance on large multi-modal models. 2024. http://apps.who.int/bookorders (24 May 2024, date last accessed).

[27] OpenAI. OpenAI | Creating safe AGI that benefits all of humanity. 2024. https://openai.com/ (24 May 2024, date last accessed).

[28] White J , Fu Q, Hays S et al A prompt pattern catalog to enhance prompt engineering with ChatGPT. 2023. https://arxiv.org/abs/2302.11382 (24 May 2024, date last accessed).

[29] Amatriain X. Prompt design and engineering: introduction and advanced methods. 2024. https://arxiv.org/abs/2401.14423 (24 May 2024, date last accessed).

[30] Bsharat SM , Myrzakhan A, Shen Z. Principled instructions are all you need for questioning LLaMA-1/2, GPT-3.5/4. 2024. https://arxiv.org/abs/2312.16171 (24 May 2024, date last accessed).

[31] Sahoo P , Singh AK, Saha S, Jain V, Mondal S, Chadha A. A systematic survey of prompt engineering in large language models: techniques and applications. 2024. https://arxiv.org/abs/2402.07927 (24 May 2024, date last accessed).

[32] Chen B , Zhang Z, Langrené N, Zhu S. Unleashing the potential of prompt engineering in large language models: a comprehensive review. 2023. https://arxiv.org/abs/2310.14735 (24 May 2024, date last accessed). 10.1016/j.patter.2025.101260 PMC12191768 40575123

[33] Zaghir J , Naguib M, Bjelogrlic M, Névéol A, Tannier X, Lovis C. Prompt engineering paradigms for medical applications: scoping review and recommendations for better practices. 2024. https://arxiv.org/abs/2405.01249 (24 May 2024, date last accessed). 10.2196/60501 PMC11422740 39255030

[34] Venerito V , Lalwani D, Del Vescovo S, Iannone F, Gupta L. Prompt engineering: the next big skill in rheumatology research. Int J Rheum Dis 2024;27:e15157. 38720410 10.1111/1756-185X.15157

[35] Kaddour J , Harris J, Mozes M, Bradley H, Raileanu R, McHardy R. Challenges and applications of large language models. 2023. https://arxiv.org/abs/2307.10169 (24 May 2024, date last accessed).

[36] Huang L , Yu W, Ma W et al A survey on hallucination in large language models: principles, taxonomy, challenges, and open questions. 2023. https://arxiv.org/abs/2311.05232 (24 May 2024, date last accessed).

[37] Tonmoy SMTI , Zaman SMM, Jain V et al A comprehensive survey of hallucination mitigation techniques in large language models. 2024. https://arxiv.org/abs/2401.01313 (24 May 2024, date last accessed).

[38] Fleming SL , Lozano A, Haberkorn WJ et al MedAlign: a clinician-generated dataset for instruction following with electronic medical records. 2023. https://arxiv.org/abs/2308.14089 (24 May 2024, date last accessed). 10.1609/aaai.v38i20.30205 PMC12826664 41584261

[39] Zhang X , Tian C, Yang X, Chen L, Li Z, Petzold LR. AlpaCare: instruction-tuned large language models for medical application. 2024. https://arxiv.org/abs/2310.14558 (24 May 2024, date last accessed).

[40] Pal A , Umapathi LK, Sankarasubbu M. MedMCQA: a large-scale multi-subject multi-choice dataset for medical domain question answering. In: Flores G, Chen GH, Pollard T, Ho JC, Naumann T, eds. Proceedings of the Conference on Health, Inference, and Learning. New York, New York, USA: PMLR, 2022: 248–60. https://proceedings.mlr.press/v174/pal22a.html.

[41] Zhao J. LLMDataHub: awesome datasets for LLM training. GitHub repository, 2023.

[42] Liu Y , Cao J, Liu C, Ding K, Jin L. Datasets for large language models: a comprehensive survey. 2024. https://arxiv.org/abs/2402.18041 (24 May 2024, date last accessed).

[43] Team G , Anil R, Borgeaud S et al Gemini: a family of highly capable multimodal models. 2024. https://arxiv.org/abs/2312.11805 (24 May 2024, date last accessed).

[44] Anthropic. The Claude 3 Model Family: Opus, Sonnet, Haiku Anthropic. https://docs.anthropic.com/ (24 May 2024, date last accessed).

[45] Singhal K , Tu T, Gottweis J et al Towards expert-level medical question answering with large language models. 2023. https://arxiv.org/abs/2305.09617 (24 May 2024, date last accessed). 10.1038/s41591-024-03423-7 PMC11922739 39779926

[46] Tu T , Palepu A, Schaekermann M et al Towards conversational diagnostic AI. 2024. http://arxiv.org/abs/2401.05654 (24 May 2024, date last accessed).

[47] Perens B , others. The open source definition. Open sources: voices from the open source revolution. O’Reilly Media, Inc, 1999;1:171–88.

[48] Touvron H , Lavril T, Izacard G et al LLaMA: open and efficient foundation language models. 2023. https://arxiv.org/abs/2302.13971 (24 May 2024, date last accessed).

[49] Liu Z , Qiao A, Neiswanger W et al LLM360: towards fully transparent open-source LLMs. 2023. https://arxiv.org/abs/2312.06550 (24 May 2024, date last accessed).

[50] Penedo G , Malartic Q, Hesslow D et al The RefinedWeb dataset for Falcon LLM: outperforming curated corpora with web data, and web data only. 2023. https://arxiv.org/abs/2306.01116 (24 May 2024, date last accessed).

[51] Zheng L , Chiang WL, Sheng Y et al Judging LLM-as-a-judge with MT-Bench and Chatbot Arena. 2023. https://arxiv.org/abs/2306.05685 (24 May 2024, date last accessed).

[52] Zhang D , Yu Y, Li C et al MM-LLMs: recent advances in multimodal large language models. 2024. https://arxiv.org/abs/2401.13601 (24 May 2024, date last accessed).

[53] Zhou H , Liu F, Gu B et al A survey of large language models in medicine: progress, application, and challenge. 2024. https://arxiv.org/abs/2311.05112 (24 May 2024, date last accessed).

[54] Acikgoz EC , İnce OB, Bench R et al Hippocrates: an open-source framework for advancing large language models in healthcare. 2024. https://arxiv.org/abs/2404.16621 (24 May 2024, date last accessed).

[55] Labrak Y , Bazoge A, Morin E, Gourraud PA, Rouvier M, Dufour R. BioMistral: a collection of open-source pretrained large language models for medical domains. 2024. http://arxiv.org/abs/2402.10373 (24 May 2024, date last accessed).

[56] Chen Z , Cano AH, Romanou A et al MEDITRON-70B: scaling medical pretraining for large language models. 2023. https://arxiv.org/abs/2311.16079 (24 May 2024, date last accessed).

[57] Ankit Pal Pasquale Minervini AGMAPG, Alex B. openlifescienceai/open_medical_llm_leaderboard. 2024.

[58] Lai VD , Ngo NT, Veyseh AB et al ChatGPT beyond English: towards a comprehensive evaluation of large language models in multilingual learning. 2023. https://arxiv.org/abs/2304.05613 (24 May 2024, date last accessed).

[59] Kianian R , Sun D, Crowell EL, Tsui E. The use of large language models to generate education materials about uveitis. Ophthalmol Retina 2024;8:195–201. 37716431 10.1016/j.oret.2023.09.008

[60] Williams CYK , Zack T, Miao BY et al Use of a large language model to assess clinical acuity of adults in the emergency department. JAMA Netw Open 2024;7:e248895. 38713466 10.1001/jamanetworkopen.2024.8895 PMC11077390

[61] Masanneck L , Schmidt L, Seifert A et al Triage performance across large language models, ChatGPT, and untrained doctors in emergency medicine: comparative study. J Med Internet Res 2024;26:e53297. 38875696 10.2196/53297 PMC11214027

[62] Chary M , Parikh S, Manini AF, Boyer EW, Radeos M. A review of natural language processing in medical education. West J Emerg Med 2019;20:78–86. 30643605 10.5811/westjem.2018.11.39725 PMC6324711

[63] McMaster C , Bird A, Liew DFL et al Artificial intelligence and deep learning for rheumatologists. Arthritis Rheumatol 2022;74:1893–905. 35857865 10.1002/art.42296 PMC10092842

[64] Zhao SS , Hong C, Cai T et al Incorporating natural language processing to improve classification of axial spondyloarthritis using electronic health records. Rheumatology (Oxford) 2020;59:1059–65. 31535693 10.1093/rheumatology/kez375 PMC7850056

[65] Love TJ , Cai T, Karlson EW. Validation of psoriatic arthritis diagnoses in electronic medical records using natural language processing. Semin Arthritis Rheum 2011;40:413–20. 20701955 10.1016/j.semarthrit.2010.05.002 PMC3691811

[66] van Leeuwen JR , Penne L, Rabelink T, Knevel R, Teng YKO. Using an artificial intelligence tool incorporating natural language processing to identify patients with a diagnosis of ANCA-associated vasculitis in electronic health records. Comput Biol Med 2024;168:107757. 38039893 10.1016/j.compbiomed.2023.107757

[67] Humbert-Droz M , Izadi Z, Schmajuk G et al Development of a natural language processing system for extracting rheumatoid arthritis outcomes from clinical notes using the National Rheumatology Informatics System for Effectiveness Registry. Arthritis Care Res (Hoboken) 2023;75:608–15. 35157365 10.1002/acr.24869

[68] Román Ivorra JA , Trallero-Araguas E, Lopez Lasanta M et al Original research: Prevalence and clinical characteristics of patients with rheumatoid arthritis with interstitial lung disease using unstructured healthcare data and machine learning. RMD Open 2024;10:3353. 10.1136/rmdopen-2023-003353 PMC10836356 38296310

[69] England BR , Roul P, Yang Y et al Extracting forced vital capacity from the electronic health record through natural language processing in rheumatoid arthritis-associated interstitial lung disease. Pharmacoepidemiol Drug Saf 2024;33:e5744. 38112272 10.1002/pds.5744 PMC10872496

[70] Lin C , Karlson EW, Dligach D et al Automatic identification of methotrexate-induced liver toxicity in patients with rheumatoid arthritis from the electronic medical record. J Am Med Inform Assoc 2015;22:e151–61. 25344930 10.1136/amiajnl-2014-002642 PMC5901122

[71] Osborne JD , Booth JS, O’Leary T et al Identification of gout flares in chief complaint text using natural language processing. AMIA Annu Symp Proc 2021;2020:973–82. 33936473 PMC8075438

[72] Venerito V , Puttaswamy D, Iannone F, Gupta L. Large language models and rheumatology: a comparative evaluation. Lancet Rheumatol 2023;5:e574–8. 38251480 10.1016/S2665-9913(23)00216-3

[73] Madrid-García A , Rosales-Rosado Z, Freites-Nuñez D et al Harnessing ChatGPT and GPT-4 for evaluating the rheumatology questions of the Spanish access exam to specialized medical training. Sci Rep 2023;13:22129. 38092821 10.1038/s41598-023-49483-6 PMC10719375

[74] Krusche M , Callhoff J, Knitza J, Ruffer N. Diagnostic accuracy of a large language model in rheumatology: comparison of physician and ChatGPT-4. Rheumatol Int 2024;44:303–6. 37742280 10.1007/s00296-023-05464-6 PMC10796566

[75] Jia F , Liu X, Deng L, Gu J, Pu C, Bai T et al OncoGPT: a medical conversational model tailored with oncology domain expertise on a large language model meta-AI (LLaMA). 2024. https://arxiv.org/abs/2402.16810 (24 May 2024, date last accessed).

[76] Madrid-García A , Merino-Barbancho B, Freites-Núñez D et al From web to rheumaLpack: creating a linguistic corpus for exploitation and knowledge discovery in rheumatology. Comput Biol Med 2024;179:108920. 10.1016/j.compbiomed.2024.108920 39047506

[77] Patel V , Stewart D, Horstman MJ. E-consults: an effective way to decrease clinic wait times in rheumatology. BMC Rheumatol 2020;4:54–6. 33073171 10.1186/s41927-020-00152-5 PMC7556892

[78] Malcolm EJ , Brandon Z, Wilson LE et al eConsults’ impact on care access and wait times in rheumatology. J Clin Rheumatol 2022;28:147–54. 35067514 10.1097/RHU.0000000000001825

[79] Ong JCL , Chang SYH, William W, Butte AJ, Shah NH, Chew LST et al Ethical and regulatory challenges of large language models in medicine. Lancet Digit Health 2024;6:e428–32. 38658283 10.1016/S2589-7500(24)00061-X

[80] Stanford University Human-Centered AI Institute. AI Index Report 2024. 2024. https://aiindex.stanford.edu/report/ (24 May 2024, date last accessed).

[81] Guidance WHO. Ethics and governance of artificial intelligence for health. World Health Organization, 2021.

[82] Meskó B , Topol EJ. The imperative for regulatory oversight of large language models (or generative AI) in healthcare. NPJ Digit Med 2023;6:120. 37414860 10.1038/s41746-023-00873-0 PMC10326069

[83] Li H , Moon JT, Purkayastha S et al Ethics of large language models in medicine and medical research. Lancet Digit Health 2023;5:e333–5. 37120418 10.1016/S2589-7500(23)00083-3

[84] Harrer S. Attention is not all you need: the complicated case of ethically using large language models in healthcare and medicine. EBioMedicine 2023;90:104512. 36924620 10.1016/j.ebiom.2023.104512 PMC10025985

[85] Gibney E. What the EU’s tough AI law means for research and ChatGPT. Nature 2024;626:938–9. 38366218 10.1038/d41586-024-00497-8

[86] Koller D , Beam A, Manrai A et al Why we support and encourage the use of large language models in *NEJM AI* submissions. NEJM AI 2024;1:AIe2300128.

[87] Fogo AB , Kronbichler A, Bajema IM. AI’s threat to the medical profession. JAMA 2024;331:471–72. 38241042 10.1001/jama.2024.0018

[88] Au Yeung J , Kraljevic Z, Luintel A et al AI chatbots not yet ready for clinical use. Front Digit Health 2023;5:1161098. 37122812 10.3389/fdgth.2023.1161098 PMC10130576

[89] Cacciamani GE , Eppler MB, Ganjavi C, Pekan A, Biedermann B, Collins GS et al Development of the ChatGPT, generative artificial intelligence and natural large language models for accountable reporting and use (CANGARU) guidelines. 2023. https://arxiv.org/abs/2307.08974 (24 May 2024, date last accessed).

[90] Luo X , Estill J, Chen Y. The use of ChatGPT in medical research: do we need a reporting guideline? Int J Surg 2023;109:3750–1. 37707517 10.1097/JS9.0000000000000737 PMC10720843

[91] Huo B , Cacciamani GE, Collins GS et al Reporting standards for the use of large language model-linked chatbots for health advice. Nat Med 2023;29:2988. 37957381 10.1038/s41591-023-02656-2

[92] Miao BY , Chen IY, Williams CYK, Davidson J, Garcia-Agundez A, Sun H et al Updating the Minimum Information about CLinical Artificial Intelligence (MI-CLAIM) checklist for generative modeling research. 2024. https://arxiv.org/abs/2403.02558 (24 May 2024, date last accessed).

[93] PlusDocs. The most overused ChatGPT words. 2023. https://www.plusdocs.com/blog/the-most-overused-chatgpt-words (24 May 2024, date last accessed).

[94] Frontiers Editorial Office . Retraction: cellular functions of spermatogonial stem cells in relation to JAK/STAT signaling pathway. Front Cell Dev Biol 2024;12.

[95] Cabanac G. Retraction Watch: signs of undeclared ChatGPT use in papers mounting. 2023. https://retractionwatch.com/2023/10/06/signs-of-undeclared-chatgpt-use-in-papers-mounting/ (24 May 2024, date last accessed).

[96] Rezaei M , Salehi H, Tabatabaei O. Uses and Misuses of ChatGPT as an AI-Language Model in Academic Writing. In: 2024 10th International Conference on Artificial Intelligence and Robotics (QICAR). IEEE, 2024: 256–60.

[97] Abbas M. Uses and misuses of ChatGPT by academic community: an overview and guidelines. SSRN 4402510. 2023. https://ssrn.com/abstract=4402510 (24 May 2024, date last accessed).

[98] Gartner. What’s new in artificial intelligence from the 2023 Gartner Hype Cycle. 2023. https://www.gartner.com/en/articles/what-s-new-in-artificial-intelligence-from-the-2023-gartner-hype-cycle (24 May 2024, date last accessed).

